# State of Lunatic Asylums and the Insane in Ireland
*"Seventh Report on the District Criminal, and Private Lunatic Asylums in Ireland." Dublin. 1855.


**Published:** 1855-10-01

**Authors:** 


### THE JOURNAL

OF

### PSYCHOLOGICAL MEDICINE

AND

### MENTAL PATHOLOGY.

OCTOBER 1, 1855.

### STATE OF LUNATIC ASYLUMS AND THE INSANE IN IRELAND.*

We have before us the seventh report of the Inspectors of Lunatic Asylums in Ireland, embodying an elaborate account of the state of the various institutions established in that country, private and public, for the care and treatment of the insane. This public document appears to have been drawn up with great care, and redounds highly to the credit of the Inspectors appointed by Government to protect the interests of the insane, and to regulate and control the various institutions devoted to their care and treatment. According to the official returns, which are made up to the 31st of March, 1855, the number of insane persons brought within the cognizance of the Inspectors, was 13,493. They are thus distributed.

| | Males. | Females. | Total. |
|---|---|---|---|
| 1. In District Lunatic Asylums . . | 1720 | 1802 | 3522 |
| 2. In Central do. . . | 84 | 42 | 126 |
| 3. In Private do. . . | 252 | 207 | 459 |
| 4. In Gaols, under 1 Vic., c. 27, and 8 & 9 Vic., c. 107 . . . . . . | 101 | 55 | 156 |
| 5. In Poorhouses . . . . . . . . | 734 | 1266 | 2000 |
| Total number of Lunatics under Official Supervision on 31st March, 1855 . . . . . . . | 2891 | 3372 | 6263 |
| 6. At large, unprovided with asylum accommodation, on 31st March, 1855 | 4035 | 3195 | 7230 |
| Total number of insane of every denomination in Ireland . . | 6926 | 6567 | 13,493 |

* "Seventh Report on the District Criminal, and Private Lunatic Asylums in Ireland." Dublin. 1855.

There appears to have been, since the Report of 1851, a decrease in the number of the insane, to the extent of 1605. This is attributable to the decrease of insane in poorhouses and in gaols. In 1851 there were confined in poorhouses 2393 lunatics; in 1855 the number was reduced to 2000. In 1851 there were upwards of 500 lunatics in the Irish gaols, whilst in 1855 there were only 156 in custody. This number will be still further reduced, in proportion to the extension of additional accommodation for the insane in public institutions, especially erected for their treatment. The Inspectors have always entertained strong objections to the residence of insane persons in such places, where they must of necessity, considerably interfere with the discipline and order which it is desirable to maintain amongst prisoners. Of the lunatics in poorhouses, nearly two-thirds are of the female sex. The Inspectors entertain grave objections to the residence of the insane in poorhouses, justly observing, that

"It is obvious, for many reasons, that the most suitable place for every demented person, lunatic or idiot, harmless or otherwise, is an institution specially devoted to the care of the insane, under the superintendence and management of experienced officers and attendants, who are practically acquainted with the treatment of mental disease in every form, and directed and controlled by that department of the public service to which the supervision of all matters relating to such establishments properly belongs; and we regard the question as deserving the consideration of the executive, namely, whether the time may not have arrived for making provision for the complete separation of the insane poor of every class, from the sane portion of the community; and which, whilst effecting a moral duty towards the latter, would insure for the insane poor, idiotic or imbecile, more care and comfort than they can possibly have in ordinary workhouses, where they cannot at all times be secured against annoyance from the ignorant or thoughtless paupers by whom they are surrounded. We feel that objections to a change may be advanced on financial grounds, and that it may be argued, considering the extremely low position which, particularly the idiotic, inmates of poorhouses occupy in the human family, both socially and mentally, that they are comfortably circumstanced and sufficiently well cared for at present.

"It is evident, however, that the attention and care necessary for the relief of these distressed classes cannot be efficaciously extended to them whilst they are placed in institutions of a very different nature from asylums; and further, it would be falling into a great mistake to imagine that even the most miserable objects of mental incapability are beyond the reach of being relieved; for there is no species of disease or affection, from the highest state of maniacal excitement to the very lowest grade of imbecility, that does not admit of cure or alleviation under judicious treatment, such alone as can be obtained in establishments exclusively devoted to the object."

To meet such a contingency the Inspectors propose enlarging some of the existing district asylums, by the erection, at a small expenditure, of suitable auxiliary buildings, with large dormitories on the ground-floor. We consider this suggestion practicable, and entitled to serious consideration. The expenditure of Asylums has, as might be naturally supposed, from the high price of provisions, greatly exceeded that of former years. The subjoined extract from the report places this matter in a clear light:—

"The average cost of maintenance, including every expense, was therefore, as above, 17*l.* 9*s.* 4*d.* for the year ending 31st March, 1854, and 19*l.* 15*s.* 10*d.* for the year ending 31st March, 1855, showing a difference of 2*l.* 6*s.* 6*d.* per head in favour of the former year.

"As a general rule, the Asylums containing most inmates exhibit the lowest averages, as Ballinasloe, Belfast, Cork, and Limerick, which show for the first year, conjointly, an average of 15*l.* 16*s.* 11*d.*, and for the second, 18*l.* 10*s.* 5½*d.* Richmond Asylum, however, the largest institution of the kind in Ireland, is an exception to the rule, the averages for the two years, respectively, being 17*l.* 4*s.* 11*d.* and 21*l.* 17*s.* 4*d.*; but the highest prices at which the necessary commodities of life generally range in the metropolis, will sufficiently account for this difference.

"The length of time the Asylums have been established appears also to exercise a beneficial influence upon the expenditure, the Armagh and Waterford (the two smallest District Asylums in Ireland, and which have been in operation—the former for thirty, and the latter for twenty years) showing, comparatively, very low averages, while those lately occupied, as Kilkenny, Killarney, and Omagh, give the highest averages of all, owing, in a great measure, to incidental expenses attendant on their opening.

"The Asylum at Cork is the only one in which no material change has occurred, the increase during the year ending 31st March, 1855, being but 3*s.* 4*d.* per head per annum on the outlay of the preceding year, while the increase in the other establishments varies from 1*l.* to 4*l.*, Londonderry alone excepted, where a diminution has taken place in 1855. This diminution is, however, accounted for by the fact that the Omagh patients were taken away in 1854, thus leaving the expenditure for that year to be distributed over a reduced number of inmates.

"Looking to Ballinasloe, Belfast, Cork, Limerick, Londonderry, and Richmond Asylums, which taken together afford an average of 325 inmates each, for the year ending 31st March, 1855, the annual outlay in salaries and wages is but 3*l.* 11*s.* per head, while in the remaining eight Asylums, which only give an average of 150 patients, we have it raised to 5*l.* 13*s.* 6*d.* The advantage which the larger institutions thus obtain in an economical point of view, is, as regards the staff, very important.

"The several averages on Diet alone range in 1854 from about 5*l.* to 7*l.*, and in 1855 from about 7*l.* to 9*l.*; but in the clothing there is very little difference between the two years, in 1854 it was 1*l*. 4*s*. 8*d*. and in 1855, it was 1*l*. 2*s*. 9*d*. per head."

In reference to the number of admissions, it appears that the inmates of the several district asylums on the 31st March, 1855, amounted to 3,299; the number being at the corresponding period of our last Report, 2870; or, 1447 males, and 1423 females; thus showing an increase of 429 within two years, namely, 205 males, and 224 females; and it is satisfactory to find that as facilities for early admission have been extended by means of the new district asylums which have lately come into operation, proportionate advantages have been obtained both in a curative and sanitary point of view, as will appear from the following summary, in which the admissions, discharges, and deaths, that have occurred during the years 1853-4, and 1854-5, are contrasted with those of the two previous years. With regard to the admissions, it should be borne in mind that two new asylums, viz., Kilkenny and Killarney, were opened in the year 1852-3, which circumstance, by adding to the admissions for the last two years ending 31st March, 1853, to an unusual extent, renders the contrast in favour of the period of which we are treating less remarkable than it otherwise would be.

| | Two Years ending 31st of March, 1853. | | | Two Years ending 31st of March, 1855. | | |
|---|---|---|---|---|---|---|
| | Males. | Females. | Total. | Males. | Females. | Total. |
| Admitted | 1064 | 1039 | 2103 | 1197 | 1114 | 2311 |
| Discharged during the two years—Cured. | 409 | 406 | 815 | 458 | 421 | 879 |
| " Relieved. | 99 | 116 | 215 | 147 | 106 | 253 |
| " Not cured | 100 | 85 | 185 | 79 | 78 | 157 |
| " Incurable | 34 | 78 | 32 | 40 | 28 | 68 |
| Total Discharged | 642 | 645 | 1287 | 724 | 633 | 1357 |
| Died during the two years | 277 | 253 | 530 | 268 | 257 | 525 |
| Number of Inmates on 31st March, exclusive of Island-bridge | 1447 | 1423 | 2870 | 1652 | 1647 | 3299 |

The admissions, according to the foregoing, during the two years ended 31st March, 1855, exceeded the admissions of the two former years by 208, namely, 133 males, and 75 females. The excess of recoveries was 64; or, 49 males and 15 females. The number discharged cured being 879, and relieved, 253, which, taken together, give a per centage of 49 on the admissions; while the mortality, notwithstanding the increase of inmates, is less by 5 than in the two former years; being a little more than 10 per cent. on the total number under treatment.

Great attention appears to have been paid to providing suitable occupations for the insane, and that with the best results. There were employed on a given day, which we have taken as affording a comprehensive view of the state of the asylums in this respect, 1891 persons, of whom 962 were males and 929 females. Of the males, 441 were engaged in gardening and agricultural labour; 83 in various trades, as weaving, shoemaking, tailoring, &c.; and 438 in a variety of miscellaneous employments. Of the females, 537 were occupied in needlework, spinning, knitting, quilting, and fancy work; 134 in the laundries; 172 in house cleaning; and 89 in miscellaneous employments.

The proportion of employed to the whole number under treatment is 57 per cent.; a very favourable proportion, when it is remembered that of the 3282 patients, 2072 were probably incurable, and 366 epileptics and idiots.

The Inspectors speak highly of the benefits which have resulted to the insane from bringing them within the sphere of religious instruction. A large majority of the patients continue to attend the chapels of the asylums on each Sunday, and by their calm and collected demeanour during Divine service, evince a sense of the purpose for which they are assembled; and it is not alone on the Sundays but during the week many of them look forward to the visits of the chaplains with the greatest anxiety.

On this point the Inspectors observe, that,

"In the treatment of mental disease, our continued experience bears us out in the opinion 'that a very large proportion of the inmates of an hospital for the insane, are capable of deriving advantages from that form of religious observance in which they have been brought up; and that hence it becomes an obvious duty to provide the necessary means of public worship in every lunatic asylum.' No doubt, in his general ministrations, and still more in his private communications, the chaplain of an asylum should not lose sight of the characters of his hearers, nor of the great judgment and delicacy called for in their regard. But it is not the patients alone who feel the important advantages of the services of ministers of religion in these establishments; for it should be recollected that the instruction imparted by them, and the performance of divine service, cannot fail to produce a marked moral benefit on the attendants also, who may be considered, from the nature of their duties, as in general debarred from a due and regular attendance at their respective places of worship on Sundays and other days of devotion."

We are gratified to observe that the Central Asylum erected at

Dundrum for the safe custody of insane persons charged with offences in Ireland, has from its opening proved eminently successful. The number of patients confined in the Asylum amounts to 126. The admissions are restricted to cases of a grave character, or to those where, though the offences might not be very serious in themselves, the offenders had evinced particularly dangerous symptoms or inveterate propensities of a criminal nature.

In reference to the general state of the criminal inmates of the Central Lunatic Asylum, it appears that 28, or nearly one-fourth of the whole number of patients, are pronounced recovered or convalescent, 15 of whom have become so within the last two years.

"Had these twenty-eight been ordinary inmates of an asylum, they would have been set free, each after a probation of about six weeks; but in the Dundrum Asylum their sojourn is much more lengthened and indefinite, as even under the most favourable circumstances we would not submit the proposition of a discharge without an unbroken restoration of mind, coupled with exemplary good conduct, for a twelvemonth, as the very shortest period."

We have in previous numbers of this Journal contrasted the state of the law with regard to the confinement and liberation of criminal lunatics in this country to that liberal, enlightened, and philosophic mode of procedure adopted so successfully in the sister country. In England, in criminal cases, an acquittal on the ground of insanity is tantamount to perpetual imprisonment, and imprisonment too under the most degrading, humiliating, and painful circumstances. Whilst under the affliction of dire disease, destroying all power of rational thought and healthy self-control, and that, too, when the mind is often tortured by wild and terrible phantasies, an overt act of crime is committed. Insanity is urged as an extenuating plea,—the jury fully recognising the irresponsibility of the prisoner, acquit him of the charge, and a verdict of "not guilty, on the ground of insanity," is properly recorded. The unhappy lunatic, who is no more accountable for his criminal act than a man would be for the wanderings of his intellect whilst under the influence of a disturbed dream, instead of being transferred, after acquittal, to the kind care of his relations and friends, is, like a common felon, handed over to a public officer, and forthwith sent to the criminal department of one of our public Asylums, there to spend the remainder of his wretched days as the miserable companion of idiots, and as the associate of the worst class of insane criminals. His attack of insanity, provocative of the offence for which he was tried, may have been temporary and transient in its character, similar, for illustration, to that of puerperal mental derangement. The law, however, in its profound wisdom, recognises amongst criminal lunatics, no distinction of classes. A man once criminally insane, continues so for the term of his natural life. A recovery is viewed as an impossibility, and liberation from restraint highly dangerous to the safety and welfare of society. The law, owing to its harshness in this particular, often stultifies itself. Consider, for instance, a case of recent occurrence, which has, unhappily for all parties concerned, obtained a painful notoriety. An amiable and intellectual lady, much beloved and highly respected in private life, the wife of a distinguished physician, in a paroxysm of obvious and unmistakeable monomaniacal delirium, abstracts from a shop an article of insignificant value. The facts connected with the offence, as well as the previous history of the mental condition of the party, clearly and conclusively demonstrated, beyond the possibility of doubt, the existence of a morbid state of the mind at the time of the commission of the alleged criminal act. This was apparent to all the medical men who were consulted, as well as to the eminent counsel employed to conduct her defence. No rational or right-thinking person at all cognisant of the facts that could have been deposed to at the trial, entertained the shadow of a doubt as to the absence of all criminality, or as to the certainty of her acquittal at the hands of a British jury. Why, then, it will be asked, was not medical evidence forthcoming at this lady's trial, and the plea of insanity urged in extenuation of her offence? We will briefly answer the interrogatory. It was apparent to the able legal advisers engaged in the case, that if this unhappy lady, who, in a moment of delirious excitement, and consequent *loss of self control*, had brought herself within the jurisdiction of the law, were to escape on the plea of legal irresponsibility caused by mental derangement, she would inevitably pass from the Central Criminal Court to the dreary and desolate wards of Bethlehem Hospital, and that no efforts that might be subsequently made to effect her liberation would lead to any satisfactory result. The husband of the lady, as well as his legal advisers, had a clear perception of this painful alternative, and it was thought better to lay before the jury a clear and succint statement of the facts of the case, leaving it to their sense of justice and humanity to decide the issue. Had the state of the law not have been so manifestly defective, another course would have been pursued, but it was thought safer to run the risk of a conviction, with its accompanying obloquy and punishment, than urge as an excuse the plea of insanity, and thus incur the danger of perpetual imprisonment among the insane.* The law pretends to acquit, on the

* We copy the subjoined particulars from the police reports of the *Times* of September 5th. We are reluctant to make any observations offensive to the magistrate whose duty it was to adjudicate in the painful case referred to in the ground of irresponsibility, induced by diseased brain and disordered mind, and *yet* PUNISHES *those so acquitted with the severest penalty short of actual death upon the scaffold!* This anomalous state of the law is a disgrace to a Christian and civilized community. We freely admit that great caution should be exercised in the liberation of perabove remarks. He was at the time exposed to severe animadversion for his alleged harsh and somewhat Spartan mode of dealing with the case. Influenced, we have no doubt, by a nervous anxiety to administer equal justice to rich and poor, he somewhat strained his judicial functions, and instead of adopting the *humane* view of the lamentable position in which the lady had placed herself, and at once viewing the matter before him in its proper light, he, by taking the extreme course, and sending the case to trial, inflicted an amount of mental anguish upon the numerous members of a much repected family beyond the reach of all remedy. Let our readers contrast this proceeding with the benevolent and enlightened course pursued by Mr. Jardine in the case that came judicially before him.

"BOW-STREET.—Jane Moseley, a young lady residing at 10, Mornington-place, Hampstead-road, was charged before Mr. Jardine with stealing a papier maché portfolio, worth 5*s.*, the property of Lavinia Price, of 18, Hart-street, Bloomsbury-square.

"Ellen Woodward stated that she was a housemaid in the service of the prosecutrix. On Monday evening the prisoner called at the residence of her mistress, and requested to see the first-floor apartments, which were to be let furnished. Witness showed her up into the drawing-room, upon which the prisoner requested her to fetch a glass of water. Witness ran down stairs for this purpose, and while she was returning with it saw the prisoner in the act of leaving the house. The circumstance having excited her suspicion, she hastened back to the drawing-room to see if anything had been removed, and, missing the portfolio from the table, followed the prisoner into the street, and accused her of stealing it. The prisoner denied the charge, but witness insisted on bringing her back to the house. The prisoner then produced the portfolio from under her shawl, and offered witness 5*s.* not to say anything about it. A policeman was called, however, and she was given into custody.

"The prisoner sobbed bitterly during the examination of the witness, and declined to say anything to the charge.

"The mother of the prisoner, who appeared equally distressed, stated that she was a widow. She had two daughters, both of them suffering from affliction, the prisoner's sister being at the present moment in the last stage of consumption. The prisoner was eighteen years of age, and, from causes peculiar to her time of life, was subject to fits of mental aberration. At such times she could not be regarded as a responsible being.

"Mr. Jardine inquired if any medical evidence to this effect could be produced.

"The mother having replied in the affirmative, the case was put back till a later period of the day, to enable her to do so.

"A gentleman, whose name did not reach us, but who stated that he was a professional gentleman residing in Caroline-street, Bedford-square, waited subsequently upon the magistrate, and informed his worship that he had known the prisoner's family for many years. They were of the highest respectability. The prisoner, he was aware, was an occasional sufferer from illness of a peculiar kind, and at such times he had known her mind to be slightly affected.

"Mr. Jardine.—Do you speak as a friend, or have you attended her professionally?

"Witness.—I have attended her professionally.

"Mr. Jardine.—You are prepared to assure me, then, from your own personal knowledge, that her mind has been so affected at times as to make her unconscious of what she is doing?

"Witness.—Yes, at short intervals.

"Mr. Jardine.—I think this is a case, then, in which I ought to restore the prisoner to her friends. She may be given up to her mother.

"The prisoner was accordingly discharged."

sons tried for capital offences, but is there not, we ask, a large number of persons at this moment confined in the criminal wards of our public Lunatic Asylums for the commission of trivial offences against the law, who might be either safely liberated from all restraint or be subjected to a modified and less offensive kind of surveillance?

But to return to the Report. When speaking of the liberation of homicidal cases, the Inspectors observe, that,

"One patient alone, a respectable married female, who destroyed her infant whist labouring under puerperal mania, has been liberated since the date of our last Report. We shall have occasion, however, in the course of the present year, to lay before his Excellency the Lord Lieutenant, for his consideration, seven or eight cases as fit subjects for freedom. Of these cases, three were acquitted of homicide; but being now for over four years under our immediate supervision, and certified by the attendant physicians to be free of every symptom of mental derangement, at the same time that they have been uniformly quiet, industrious, and well conducted, we feel justified in the course we propose—the more so as they undertake to emigrate; two having already received money for the passage out to join their families.

"Independent of the exercise of clemency itself, in a moral point of view, the very fact of opening the gates of an asylum such as the Dundrum, and affording egress to objects deemed worthy of it, produces a beneficial and tranquillizing influence over those who remain behind, and who, if finding no prospective hope of freedom on recovery, but obliged to regard their future doom as the companions of madmen, might, from their very numbers, become most dangerous and difficult to control. There are two individuals, both respectably connected, acquitted of very aggravated assaults, and who being now, and indeed since their transference to the asylum, quite well, might be liberated; but as the parties on whom the assaults were committed (one the father, the other a solicitor) object to their enlargement, in deference to strong personal apprehensions, and aware of the responsibility we might incur if any thing untoward subsequently took place, we are unwilling to interfere. In the course of time, however, should those justifiable fears subside, or if some arrangement can be effected by us between the various parties, we trust they may then participate in the same consideration extended by Government to others.

In alluding to the importance of inquiring into the antecedents of criminal lunatics, and suggesting modifications of the law with regard to them, the Inspectors observe:—

"It would be very desirable in all important cases, as when a party is acquitted, on the plea of lunacy, of murder, or of a serious attempt on the life of another, that the antecedents to the act should at the time be judicially investigated. Once insanity is established—and it generally happens to be the first point urged in defence—the case closes; and those exciting causes, or incidental circumstances, likely to modify the judgment of the court in regard to a prisoner thoroughly responsible for his conduct, are left unquestioned. Thus the lunatic labours under a disadvantage in one respect; for though acquitted of a moral crime, he may still become the penal sufferer by a more lengthened confinement. Amongst other instances under our cognizance, as illustrative of this view, we shall refer to three: the first, that of a man in the Central Asylum, who it was proved, whilst labouring under maniacal excitement from jealousy towards his wife in consequence of her supposed freedom of conduct, committed homicide. On recovery from his insanity he was brought to trial, when the fact was proved. This person is now quite sane, and has been so for some years. The second instance we may adduce in the person of a man, who, in a scuffle, inflicted a wound which ultimately caused death; he was acquitted on the plea of being deranged at the time of the occurrence. The third is one of a peculiar character, for the individual in question, acquitted also on the plea of insanity, complains that he has thereby been most unfairly and harshly treated; that he never was deranged; that the offence he committed was the result of the hardship and injustice he suffered at the hands of another, and of his consequent anger and excitement; and that had he been tried regularly, and found guilty, he would have escaped with a comparatively short imprisonment.

"The records alone of the fact of trial, and the cause of acquittal, exist in these and similar cases; but it would be most satisfactory if such records were coupled with the official information of attendant circumstances, in order that, when submitting them to the consideration of the Lord Lieutenant, we might be enabled to furnish ample materials for his Excellency's decision; and to satisfy ourselves we were justified in stating them. So long as an asylum—no matter under what denomination, be it even that of criminal—is made the receptacle of our unfortunate fellow creatures, who, in the hour of grievous mental derangement, have committed offences in themselves the most appalling, or of those who, subsequent to sentence, may lose their reason altogether, so long its inmates have a claim on our kindliest sympathies; it ceases, however, to fulfil its object if, through a mistaken benevolence, or from want of a scrutiny into the particulars of each case, it should become the residence of parties for whom it was not legitimately intended, or should an immunity for the undeserving be secured within its precincts."

Reverting to the statistics in the Report, it appears, that of the twenty-eight criminal lunatics returned as cured, twelve were homicidal cases, nine being males and three females. Up to the time of making the report the total number of homicidal cases admitted into the Dundrum Asylum, were thirty-five males and nineteen females. The most frequent kind of homicide among the men is wife murder. No less than eight committed this offence, independent of those who attempted it, but fortunately without effect. This fact, at first sight, might seem to argue less constancy, fidelity, and tenderness with the male sex; but there are strong causes to explain away, or at least reduce the force of the conclusion; for it is well known that, occasionally, among the first and most marked symptoms of the disease with lunatics, may be reckoned a mistrust and aversion to members of their own family, and to those particularly with whom they had been united by the strongest ties of affection, and who, if physically weaker, in case any control is attempted, are most exposed to suffer from their violence. On the other hand, there is no record of a female killing her husband, the most common mode of destruction among women being infanticide, of which there was lately a very melancholy instance in the Dundrum Asylum. Great commiseration is, no doubt, due to many who come within this category; for we can fully imagine how shame and anguish must weigh on an unfortunate and betrayed female, with enfeebled system, what strong temptations induce her to evade the censure of the world in the destruction of the evidence of her guilt, by a crime that outrages her most powerful instinct, maternal love of offspring. The thought of such a fearful exposure no doubt may lead to some sudden and impulsive act, for which, as generally happens, she is judged with the utmost leniency; but still, unless the deed is accompanied with, and followed by distinct symptoms of insanity, the difficult question presents itself: Is such a person—sane immediately after the act, sane at trial, and sane on admission—to remain for life—or if not for life, for what period—the inmate of an asylum, and the associate of lunatics?

The subjoined remarks, with reference to the particular tendencies of the insane, homicidal and suicidal, their fixity of delusion, responsibility, and peculiarities, will be read with great interest.

"With regard to the existence of particular fixed tendencies among the insane, we are sure it will be both agreeable and interesting to your Excellency to be informed, that although the Dundrum Asylum is specially erected for criminal lunatics, and contains so many who have deprived their fellow creatures of existence, our experience of its inmates would almost ignore propensities of a decidedly homicidal character, although there are some patients in it with marked suicidal inclinations. We are aware of three only who have evinced this destructive propensity—a convict from Spike Island, sentenced to transportation for burglary, and two men who committed murder; one of them, however, cannot be considered as innately malevolent, for he labours under a double delusion, inasmuch as he thinks if he can succeed in killing some person, he will thereby obtain his freedom here, and secure heaven hereafter. We would, therefore, in a spirit of humanity, be disposed to infer that some of the most serious offenders in the Asylum have become so more from accidental causes than from an original malignity of disposition.

"Revolving on the whole class of homicides and those who have committed dangerous assaults on the person, it is difficult to assign how far responsibility may or may not be occasionally attached to certain individual acts. When persons are thoroughly insane, it is clear they are accountable for no crime, however frightful in itself; practically, however, from experience we would conclude that lunacy has not always been so developed as to destroy every idea of right and wrong, and a consequent feeling of responsibility; whilst it may have happened in a case or two within our cognizance that insanity was assumed to escape the ends of justice.

"The difficulties thrown in the way of liberating lunatics from the Central Asylum after recovery, and the length of their subsequent detention—for it is only by warrant of the Lord Lieutenant, based upon the certificate of the physicians and the final report of the Inspectors, that they are set free—has afforded some precise information on a point which is not so satisfactorily arrived at in ordinary institutions, from which patients are promptly discharged—viz., the permanency of cure. We have several cases where the parties seemed to have recovered their reason perfectly, and relapsed. One man (a homicide) who was steady and rational for nearly three years, became, without apparent cause, suddenly and boisterously insane; and now again, at an interval of four months, is tranquil and quite collected. Another, who came in highly excited, continued so, with little variation, for two years, when he rapidly got well to all appearance, remained so about eighteen months, and is now again as bad as ever. Some continue well for months, and then relapse; and while under all the favourable circumstances which comfort, regularity, and a healthy residence can produce. A peculiarity has hitherto marked these recurrent attacks—with one exception, they have been confined to the male sex.

"Occasionally, too, it has come within our observation that a perfect restoration of the mental powers takes place, so far as can be judged by an undeviating reasonableness of conduct and conversation, although the original delusion, which led to acts the most insane, continues in full force. We gave, in a former Report, the details of a case where the captain of a ship killed seven of his crew at a short distance off Cork harbour. For twelve consecutive years, this man had periodical attacks of violent madness; within the last four, however, he has shown no symptom whatever of disease; his time is principally devoted to pious reading; though never spoken to on the subject of his offence (according to an established rule in the Asylum), still, of his own accord, he has lately expressed his conviction that the crew actually mutinied with intent to murder him. The delusion is thus as fixed as ever, and, did a second opportunity present itself, the consequences, as far as depended on him, might, under similar circumstances, be alike unfortunate.

"As may be supposed, in an institution such as the Dundrum Asylum, many of the patients are at times highly excited and intractable; others morose and reserved, as if, in the sort of twilight intelligence they retained, their minds were engrossed with one train of ideas, or some painful remembrance of the past.

"Among others we have a remarkable case of this character in a homicide, who for five years, with only three exceptions, where he suddenly made some short and angry observations, has maintained an unbroken silence; he smiles occasionally, if he hears anything amusing, but never condescends to laugh. This man will stand with his head bent to his chest for four or five hours together in the same exact position, and resist being moved simply by the vis inertiæ; yet he goes, when called, to his meals, and behaves most correctly at table. At the female side too, there is an infanticide, very taciturn and reserved, with a strong predisposition to self-destruction; her attempts are always with broken glass, which she endeavours to secrete; scissors and knives may be safely entrusted to her. A gloom hangs over this woman; her conversation is most rational—in the one object alone, and by the one mode of attaining it, is her insanity displayed. We mention these individual cases as characteristic of many, and thus illustrating to your Excellency, on subjects more or less professional, that general information which we would desire to convey.

"With the exception of two attempts at life, but neither fatal, we have had no serious cases to refer to officially since the date of our last Report. In the first instance, a lunatic inflicted a deep wound on his own neck with a razor; in the second, a convict from Spike Island, at the time deemed quite harmless (but who has since evinced a cold, malignant disposition), stealthily getting behind one of his companions, whose verses and sarcasms give occasional annoyance, struck him on the head with a piece of iron he had secreted for the purpose, causing a large compound fracture, through which a considerable quantity of brain was discharged. Dr. Harrison, the Visiting Physician, was in immediate attendance, and removed several pieces of depressed bone; after remaining comatose for four days, the man recovered rapidly, without an unfavourable symptom, and without the slightest change or remission of his insanity. By a strange coincidence this patient was noted for his memory, and the accuracy with which he repeated a long string of words the most incongruous. The wound, with a considerable loss of brain, was (phrenologically speaking) exactly in the region of the organ of memory; his recollection, however, continues quite unimpaired."

The remarks of the Inspector with regard to private asylums in Ireland we publish in another part of our Journal.

The following is a Return of Patients in Private Lunatic Asylums on 31st December, 1854, classified as to Professions, &c.

| | Males. | Females. |
|---|---|---|
| Married . . . . . . . . . . . . . | 48 | 57 |
| Single or Widowed . . . . . . . . . | 204 | 150 |
| Total. . . . . . . . . | 459 | |

PREVIOUS PROFESSIONS OR OCCUPATIONS:

| | |
|---|---|
| Army | 35 |
| Navy | 3 |
| Church | 19 |
| Law | 18 |
| Medicine | 6 |
| Students | 15 |
| In Trade | 36 |
| Other occupations | 49 |
| Farmers | 16 |
| No occupation | 272 |
| Total in Asylums | 459 |

| | Males. | Females. |
|---|---|---|
| Found Lunatic by Inquisition | 19 | 7 |
| Sent by Authority of Friends | 233 | 200 |
| Total | 459 | |

The appendix contains a vast body of interesting and important statistical and tabular statements, apparently drawn up with great care.

